# Critical Incident Reporting in Anaesthesia: A Prospective Internal Audit

**Published:** 2009-08

**Authors:** Sunanda Gupta, Udita Naithani, Saroj Kumar Brajesh, Vikrant Singh Pathania, Apoorva Gupta

**Affiliations:** 1 Professor, Department of Anaesthesia and Critical Care, RNT Medical College, Udaipur 313001, Rajasthan; 2 Assistant Professor, Department of Anaesthesia and Critical Care, RNT Medical College, Udaipur 313001, Rajasthan; 3 Medical Officer, Department of Anaesthesia and Critical Care, RNT Medical College, Udaipur 313001, Rajasthan; 4 PG Student, Department of Anaesthesia and Critical Care, RNT Medical College, Udaipur 313001, Rajasthan; 5 Sr. Resident, GBH American Hospital, Udaipur 313001, Rajasthan

**Keywords:** Critical incidents, Critical incident reporting, Human error, Mortality, Anaesthesia related mortality

## Abstract

**Summary:**

Critical incident monitoring is useful in detecting new problems, identifying ‘near misses’ and analyzing factors or events leading to mishaps, which can be instructive for trainees. This study was aimed at investigating potential risk factors and analyze events leading to peri-operative critical incidents in order to develop a critical incident reporting system. We conducted a one year prospective analysis of voluntarily reported 24- hour-perioperative critical incidents, occurring in patients subjected to anaesthesia. During a one year period from December 2006 to December 2007, 14,134 anaesthetics were administered and 112(0.79%) critical incidents were reported with complete recovery in 71.42%(n=80) and mortality in 28.57% (n=32) cases. Incidents occurred maximally in 0-10 years age (23.21%), ASA 1(61.61%), in general surgery patients (43.75%), undergoing emergency surgery (52.46%) and during day time (75.89%). Incidence was more in the operating theatre (77.68%), during maintenance (32.04%) and post-operative phase (25.89%) and in patients who received general anaesthesia (75.89%). Critical incidents occurred clue to factors related to anaesthesia (42.85%), patient (37.50%) and surgery (16.96%). Among anaesthesia related critical incidents (42.85% n=48/112), respiratory events were maximum (66.66%) mainly at induction (37.5%) and emergence (43.75%), and factors responsible were human error (85.41%), pharmacological factors (10.41%) and equipment error (4.17%). Incidence of mortality was 22.6 per 10, 000 anaesthetics (32/14,314), mostly attributable to risk factors in patient (59.38%) as compared to anaesthesia (25%) and surgery (9.38%). There were 8 anaesthesia related deaths (5.6 per 10, 000 anaesthetics) where human error (75%) attributed to lack of judgment (67.50%) was an important causative factor. We conclude that critical incident reporting system may be a valuable part of quality assurance to develop policies to prevent recurrence and enhance patient safety measures.

## Introduction

In recent years anaesthesia, in spite of low mortality, is still associated with significant morbidity. There appears to be considerable conformity that anaesthesia risk is an important public health concern and that it is reducible^1^. Further, there is reason to believe that a substantive portion of that risk is related to human error resulting from errors in management or deviation from accepted practice^2^. If the frequency of error has to be decreased, a clearer understanding of that process is needed, the circumstances that encourage error should be identified and the relative frequencies of different classes of errors should be established.

Since its early adoption in the field of aviation^3^ and later in the field of anaesthesia^4^,^5^; the collection of data on critical incidents is gaining acceptance in anaesthesia. However there are still sporadic studies^6^‐^8^ from the developing countries which have tried to analyze and evaluate the frequency of critical incidents “related” to anaesthetic procedures.

Ouraim was to identify the incidence, outcome and potential risk factors leading to critical incidents during anaesthesia in a general tertiary care teaching hospital catering to mostly tribal patients and to promote voluntary reporting of critical incidents in our department.

## Methods

After obtaining approval from the hospital ethics committee, a one year prospective analysis of perioperative critical incidents was conducted in a tertiary care teaching hospital situated in a tribal belt from December 2006 to December 2007. Since it was an observational study without any intervention, consent from patient was not required.

In a faculty meeting of the department, it was decided to implement ‘critical incident reporting’ as a quality assurance measure and anaesthesiologists were asked to report 24-hour-perioperative critical incidents, occurring in patients subjected to anaesthesia. A critical event was defined as “An event under anaesthesia care which had the potential to lead to substantial negative outcome (ranging from increased length of hospital stay to death or permanent disability or cancelled operative procedure) if left to progress”^4^,^9^

Indigenous “Critical Incident Reporting Form” was developed and were made available in all the operation theatres, post operative wards and Intensive Care Units or High Dependency Units. Anaesthesiologists were regularly motivated and reminded to report critical incidents on an anonymous and voluntary basis and care was taken to maintain complete confidentiality. In these forms, detailed contextual information during recording of an event which would enhance the subsequent review of the incident was also included.

The critical incident reporting form had two parts:

1. Description part: It was filled by anaesthesiologists who were conducting the case. Patient's age, sex, ASA grading, previous systemic involvement, emergency/elective surgery, surgical specialty, factors related to anaesthesiologist conducting the case, time, type of anaesthesia, place and phase of occurrence of critical incident, time and means of detection, type and details of systemic event and substantial negative outcome were recorded.
2. Analysis part: All completed forms of critical incidents including mortality were reviewed and analyzed by senior consultant an aesthesiologist of the department. These critical incidents were later assigned to factors attributable to either patient or anaesthesia or surgery. When only one of these factors was responsible it was defined as “totally attributable” and if patient factor was associated with either anaesthesia or surgery factor it was defined as “partially attributable” to anaesthesia and surgery respectively. Anaesthesia related critical incidents and mortality were further analyzed for factors responsible like equipment error, pharmacological factor and human error including lack of judgment, or skill, or experience and failure to check.

Data were expressed as number and proportion to calculate incidence.

## Results

During the one year study period 14,134 anaesthetics were administered and 112(0.79%) critical incidents were reported with complete recovery in 80(71.42%) and mortality in 32(28.57%) cases.

Distribution of critical incidents was almost same in males and females (49.11% and 50.89% respectively) with a maximum incidence in 0-10 year age group (23.21%). Majority of critical incidents occurred in ASA grade I patients (n=69, 61.61%) as compared to ASAII (n=27, 24.11%) III (n=15, 13.39%) and IV (n=1, 0.89%) patients. Incidence was maximum in patients with no pre-existing systemic involvement (n=69, 61.61%) followed by cardiovascular (n=19, 16.96%) and respiratory (n=8, 7.14%) involvements. Incidents were observed more between 6am to 6pm (75.89%), in emergency patients (54.46%), and in patients admitted for general surgery (43.75%), (Table 1).

**Table-1:** Distribution of critical incidents according to surgical specialty involved [Values are number(proportion)]

SNo.	Surgical specialty	No.	%
1	General surgery	49*	43.75%
2	Gynecology and Obstetrics	21	18.75%
3	Pediatric Surgery	10	8.93%
4	Neurosurgery	4	3.57%
5	Otorhinolaryngology	10	8.93%
6	Plastic	2	1.79%
7	Orthopedic	8	7.14%
8	Cardiothoracic	4	3.57%
9	Eye	2	1.79%
10	Urology	2	1.79%
11	Total	112	100.00%

* distribution of general surgery patient was as follows- • Exploratory laparo to my for intestinal obstruction or perforation peritonitis, trauma = 15, • Surgery on gallbladder, pancreas, spleen = 12, • Surgery on renal system = 8 • Hernia/hydrocele/appendicectomy = 6, • Abscess = 7 • Breast surgery = 1

**Fig 1:**
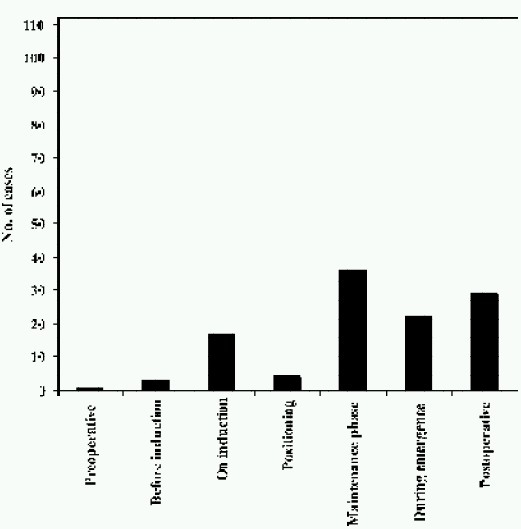
Distribution of events in perioperative period

Incidents occurred more frequently inpatients who received general anaesthesia (75.89%) with most of the incidents occurring in the operating room (77.68%) or in post-operative ward (13.39%). Critical incidents occurred most commonly during the intra operative / maintenance phase (32.04%) and frequently in the post operative period (25.89%), (Fig. 1).

Majority of these incidents (98.12%) were detected by alert anaesthesiologists either clinically (38.39%) or by monitoring equipments (23.21%) or simultaneously by both (38.39%).

In our institution, resident doctors who are under training for post graduation conduct cases under the supervision of senior consultants. Critical incidents occured in 36 cases (32.14%) which were being conducted independently by resident doctors with less than 3 years experience. In rest of the cases resident doctors were supervised by consultants with experience of 3-5 years (n=45, 40.17%) or more than 5 years (n=41, 27.67%). There was no indication of stress among the anaesthesiologists conducting the cases. All the incidents had occuned when the workload of the anaesthesiologist was less than 12 hours, without any report of contributing factors like haste, distraction or inadequate help. Most of the critical incidents were due to events involving either respiratory system (39.29%), or cardiovascular system (32.14%) or both (9.82%), (Table 2).

**Table 2:** Distribution of critical incidents according to occurrence of events.[Values are number(proportion)]

Type of event (according to System involvement) n=112	Description of event (n=112)		Cardiac arrest with out come (n=41)		
		No.(%)	Revived (n=9)	Dead (n=32)	Total (n=41)
**1)Respiratory** n=18+26=44(39.29%)					
**a)Airway** (n=18)16.07%	Laryngo spasm	11(9.82%)	1	1	3(2.68%)
	Can't ventilate	2(1.79%)		1	
	Esophageal intubation	4(3.57%)			
	Accidental extubation	1(0.89%)			
**b)Pulmonary**(n=26)23.21%	Hypoxia	12(10.71%)		2	6(5.35%)
	Bronchospasm	6(5.36%)			
	Aspiration	5(4.46%)		2	
	Incomplete reversal with early extubation-Hypoxia	1(0.89%)		1	
	Pulmonary edema	2(1.79%)		1	
**2)Cardiovascular event** (n=36)32.14%	Hypotension	22(19.64%)	2	9	21(18.75%)
	Bradycardia	2(1.79%)			
	Cardiac arrest	8(7.14%)	4	4	
	Myocardial infarction	2(1.79%)		1	
	PSVT	1(0.89%)			
	Ventricular tachycardia	1(0.89%)		1	
**3)Cardiovascular+Respiratory events** (n=11)9.82%	Hypoxia+Hypotension	5(4.46%)		4	7(6.25%)
	Hypoxia+bradycardia	2(1.79%)	2		
	Pneumothorax+Hypotension	1(0.89%)			
	Pulmonary edema+M.I.	1(0.89%)		1	
	Hypoxia+M.I.	2(1.79%)			
**4) Central nervous system** (n=1)(0.89%)	Headache	1(0.89%)			
**5)Cardiovascular+ central nervous system**(n=4)3.57%	Hypotension+Convulsion/drowsiness/Paralysis	4(3.57%)			
**6)Cardiovascular+central nervous system+respiratory**(n=4)3.57%	Hypotension+convulsion+hypoxia/altered Sensorium/numbness	4(3.57%)		1	1(0.89%)
7)MODS*(n=3)2.68%	Septicaemic shock	3(2.68%)		3	3(2.67%)
**8)Miscellaneous**(n=9)8.01%	Extravasation	1(0.89%)			
	Pruritus	1(0.89%)			
	Surgical emphysema with hypercarbia	7(6.25%)			
**Total (n=112)**		n=112	9(8.04%)	32(28.57%)	41(36.6%)

* **MODS**: Multiple organ dysfunction syndrome.

From a total of 112 reported critical incidents, cardiac arrest occurred in 41 cases (36.6%, 29 per 10,000 anaesthetics) out of which 9 cases (8.03%) recovered completely and 32(28.57%) had a fatal outcome (22.6per 10,000 anaesthetics). The occurrence of critical incidents led to postponement of surgery in only 2 cases: one occurred during induction of anaesthesia (7-year-male child posted for herniotomy under general anaesthesia had hypoxia and bradycardia during induction leading to cardiac arrest but was resuscitated with full recovery) and the other occurred during prone positioning of the patient (57 year old male posted for lumbar laminectomy had paroxysmal supraventricular tachy cardia with hypotension that responded to esmolol).

Critical incidents and mortality were correlated with factors attributable to either patient or anaesthesia or surgery. Table 3 shows that out of 112 critical incidents maximum incidents (42.86%, n=48) were related to anaesthesia factor [Totally attributable in 40.18% (n=45) and partially attributable in 2.68% (n=3)], followed closely by patient factor (37.5%, n=42). On the contrary, out of 32 mortalities 59.38%, (n=19) were due to patient's pre-existing condition. Anaesthesia factor was responsible for 25% (n=8) mortalities [Totally attributable 18.75% (n=6); partially attributable 6.25% (n=2)]. Respiratory events were responsible for most of the anaesthesia related critical incidents (n=32/48,66.66%) and mortality (n=4/8,50%), (Table 4). Human error was the most common responsible factor for anaesthesia related critical incidents (n=41/48,85.41%) and mortality (n=6/8,75%), while equipment error and pharmacologic factor were less common factors responsible, (Table 5, 6).

**Table 3:** Analysis of reasons for critical incidents and mortality [Values are number (proportion)]

Factors implicated	Critical incidents		Mortality
		n=112	n=32
Patient		42(37.50%)	19(59.38%)
Surgery	T*	19(16.96%)	3(9.38%)
	P**	3(2.68%)	2(6.25%)
Anaesthetic	T*	45(40.18%)	6(18.75%)
	P**	3(2.68%)	2(6.25%)

T* totally attributable (either patient/surgery/anaesthetic factors),

P** partially attributable (patient factor with either anaesthetic/surgery factor)

**Table-4:** Distribution of anaesthesia related critical incidents according to type and description of events (n = 48/112) [Values are number (proportion)]

Type of event (according to System involvement)	Description of event (n=48)		Cardiac arrest with outcome (n=11)		
		No.(%)	Revived	Dead	Total
**1)Respiratory** n=16+16=32(66.67%)					
**a)Airway events**(n=16)33.33%	Laryngospasm	10(20.83%)	1	1	2(4.16%)
	Can't ventilate	1(2.08%)			
	Esophageal intubation	4(8.33%)			
	Accidental extubation	1(2.08%)			
**b)Pulmonary events** (n=26)33.33%	Hypoxia	10(20.83%)		1	3(6.5%)
	Bronchospasm	2(4.17%)			
	Aspiration	2(4.17%)		1	
	Early extubation-hypoxia	1(2.08%)		1	
	Pulmonary edema	1(2.08%)			
**2)Cardiovascular events** (n=8)16.66%	Hypotension	3(6.25%)		2	5 (10.41%)
	Bradycardia	2(4.17%)			
	Cardiac arrest	3(6.25%)	2	1	
**3)Cardiovascular+ respiratory events** (n=1)2.089%	Early extubation-hypoxia+ Pulmonary edema+ myocardial infarction	1(2.089%)		1	1(2.08%)
**4)Central nervous system events**(n=1)2.089%	Headache	1(2.08%)			
**5)Cardiovascular+ central nervous system**(n=2)4.17%	Hypotension+convulsion/paralysis	2(4.17%)			
**6)Cardiovascular+central nervous system +Respiratory**(n=2)4.17%	Hypotension+ hypoxia+convulsion/Numbness	2(4.17%)			
**7)Miscellaneous** (n=2), 4.17%	Extravasation	1(2.08%)			
	Pruritus	1(2.08%)			
Total		n=48	3	8	11

**Table 5:** Analysis of anaesthesia related critical incidents (n=48/112), values are n(%)

Variables	No. of patients (n=48)			
**1)ASA status**	I(35,72.91%)			
	II(10,20.83%)			
	III(3,6.25%)			
**2)Emergency or Elective**	Elective (25,52.08%)			
	Emergency (23, 47.91%)			
**3)Previous system involved**	No system (41, 85.41%)			
	Cardiovascular system (5, 10.41%)			
	Respiratory (1, 2.08%)			
	Others (1, 2.08%)			
**4)Phase of occurrence**	Before induction (2, 4.17%)			
	On induction (18, 37.5%)			
	Positioning (2, 4.17%)			
	Maintenance (5,10.41%)			
	Emergence (16,33.33%)			
	Postoperative (5,10.41%)			
**5)Technique of anaesthesia**	General anaesthesia (37, 77.08%)			
	Spinal (6, 12.5%)			
	Epidural (2, 4.17%)			
	Combined spinal epidural (1, 2.08%)			
	Local block (1, 2.08%)			
	Regional+General anaesthesia (1,2.08%)			
**6)Place of occurrence**	Operation Theatre (44, 91.6%)			
	General ward (2, 4.17%)			
	Intensive Care Unit (2, 4.17%)			
**7)Factor responsible for incident**	i)Human error (41,85.41%)			
	Lack of skill (6,12.5%)	Lack of experience (9,18.75%)	Lack of judgment (18,37.5%)	Failure to check (8,16.66%)
	ii)Equipment error (2,4.17%)			
	iii) Pharmacological factor (5,10.41%)			

**Table-6:** Analysis of anaesthesia related mortality (n=8/112)

S.No	Variable	No. of Patients (n = 8)	
1	**ASA status**	I=(4, 50%)	
		II=(2, 25%)	
		III=(2, 25%)	
2	**Emergency/Elective**	Emergency (4, 50%)	
		Elective (4, 50%)	
3	**Pre-existing system Involvement**	No system involved (5, 62.5%)	
		Cardiovascular system (3, 37.5%)	
4	**Place of occurrence**	Operation Theatre (5, 62.5%)	
		Intensive Care Unit (2, 25%)	
		General ward (1, 12.5%)	
5	**Phase of occurrence**	Induction (1, 12.5%)	
		Positioning (1, 12.5%)	
		Maintenance (1, 12.5%)	
		Emergence (2, 25%)	
		Postoperative (3, 37.5%)	
6	**Technique of anaesthesia**	General anaesthesia (7, 87.5%)	
		Combined spinal epidural (1, 12.5%)	
7	**Type and description of Incident**	Type of event	Description
		i)Airway (1,12.5%)	Laryngospasm (1,12.5%)
		ii)Pulmonary (3,37.5%)	Early extubation – hypoxia (1, 12.5%)
			Aspiration-hypoxia (1,12.5%)
			Lack of oxygen supply-hypoxia (1, 12.5%)
		iii)Cardiac (3,37.5%)	Cardiac arrest (1, 12.5%)
			Hypotension anaphylactic shock (1, 12.5%)
			Hypotension high spinal (1,12.5%)
		iv)Cardiopulmonary (1,12.5%)	Early extubation-hypoxia Myocardial infarction and pulmonary edema (1, 12.5%)
8	**Factor responsible**	i)Human error (6,75%)	Lack of judgment (5, 67.5%)
			Failure to check (1, 12.5%)
		ii) Equipment = 0	
		iii) Pharmacological (2, 25%)	
			Anaphylaxis (1, 12.5%)
			Side–effect (1, 12.5%)

## Discussion

Internal audits based on recording of critical incidents in institutions are imperative for the speciality of anaesthesia, firstly, to study the changes in patient outcome which underline the improvement in standards of anaesthesia care and secondly, for sharing and discussing these critical incidents to evolve new policies to prevent recurrences^10^‐^13^

Many variables (patient status, surgical procedure, and surgical expertise) make the delineation of anaesthesia related factors obscure. The relative rarity of adverse outcome makes it imperative to study large number of patients over time. The methods used to collect information about safety of anaesthesia and to establish the risk factors have included peer reviews, hospital audit, reports to medical defense societies^14^, retrospective^4^ and prospective studies^15^. A prospective reporting system avoids the problems of inaccurate recall and allows warnings and advice to be issued if necessary, soon after the occurrence^15^. In our institution we conducted a prospective survey of 24-hour perioperative critical incidents over a one year period and found 112 critical incidents with over all incidence of 0.79% of which 0.33% (n=48) were attributable to anaesthesia. The frequency of incidents reported from different institutions have varied from 0.28% to 2.8%^16^,^17^ while higher incidence of 12.1%^18^ and 10.6%^19^ have also been reported. The vast difference in these figures lies in the fact that interpretation of critically ill in anaesthesia varies according to individual perception of an incident and to an ambiguity in how these are applied in practice. There is reluctance to report seemingly minor events while some major events go unreported for fear of retribution, lack of motivation and lack of acceptance of the fact that it could be beneficial as an educational tool^20^.

Recent studies define mortality associated with anaesthesia as death under, as a result of, or within 24 hour of an anaesthetic^21^,^22^. In literature, crude anaesthetic mortality (i.e. combined anaesthetic and surgical mortality) associated with anaesthesia ranges between 10-30 per 10,000 anaesthetics^23^‐^25^. It has been suggested that anaesthesia related mortality has decreased in the last three decades and currently ranges from 0.05 to 10 per 10,000^21^,^26^,^27^ and in most developed countries lies between 0.12-1.4 per 10,000 anaesthetics^28^.

In our audit, crude anaesthetic mortality was 22.6 per 10,000 and anaesthesia related mortality was 5.6 per 10,000 anaesthetics. The reasons for higher mortality rate in our audit as compared to developed countries may be due to the fact that we do not have an effective primary and secondary health care system in our country, resulting in tertiary care hospitals like ours dealing with more poorly optimized, sicker patients. Anaesthesia related mortality figures may well be different in the developing countries where only limited trained work force, monitoring and training facilities are available^25^,^29^.

Independent predictors of operative mortality cited in literature include advanced and pediatric (less than 1 year) age group as well as male gender^30^,^31^. We found no correlation between sex and occurrence of critical incidents or mortalities. There was no association of mortality with age however maximum critical incidents occurred in 0-10 year age group, which shows that the paediatric population are always at risk of anaesthesia because of anatomical and physiological reasons^18^,^28^,^32^.

In our audit, incidence of critical incidents and mortalities was maximum in ASA I and II patients, as maximum surgical patients belonged to this physical status. In higher ASA physical status senior consultant attendance, stringent monitoring and extra vigilance could be a reason for less incidence^6^,^7^. Though some authors have found a clear relationship between increasing ASA grade and the risk of critical incidents particularly physiological incidents^18^ and mortality^8^,^28^.

There has been a slightly higher incidence of critical incidents^18^ and mortalities^8^,^28^,^33^ in emergency surgery as compared to elective surgery. Poor optimization of patient's pre-operative status, non-availability of equipments, emergency drugs, investigation facilities and poor operating conditions are all contributory factors in emergency situation in the developing countries.

Critical incidents mostly occurred during the day-time^7^ co inciding with peak working hours in our institution. However it could be argued that compliance with reporting is low at late hours. General surgery patients were found more vulnerable to occurrence of critical incidents which may be due to more number of patients operated under general surgery, more chance of fluid and electrolyte imbalance and sepsis in these patients^6^,^9^

We found in common with others that the frequency of critical incidents and mortality was higher with general than neuraxial anaesthesia^6^,^28^,^31^,^33^. However this may be because many high risk surgeries are performed under general anaesthesia including cardiac, thoracic and neurosurgical procedures. Likewise there may be abias towards general anaesthesia in emergency settings or in patients with co-existing medical conditions. The most comprehensive recent survey of cardiac arrest incidence during neuraxial anaesthesia reported as 2.7 per 10,000 anaesthetics^33^ is nearly similar to our study (3.4 per 10,000). Improved knowledge of neuraxial block physiology and the use of new local anaesthetics with fewer side effects, associated with more routinely used oxygen monitoring through pulse oximetry has substantially decreased the possibility of major complications during neuraxial anaesthesia.

We found no correlation between occurrence of critical incidents and mortalities and experience level of anaesthesiologist^7^,^32^. It has been shown that fatigue adversely affects the professional performance of anaesthetists^34^. Since our resident doctors have approximately an 8 hourly work schedule with an average work force of 1-2 anaesthesiologist per case, there were no reports of stress, haste, inattention, fatigue or inadequate help as reported by other workers^32^,^35^.

Operating room was observed as a vulnerable site for occurrence of critical incidents^7^,^9^. Induction and maintenance phase have been considered as “incident rich phase”^6^,^8^ but we found a higher incidence in the maintenance and post-operative phase, probably the latter could be attributable to the inadequate post-operative monitoring and care available in our institution. However anaesthesia related incidents occurred maximally during emergence and induction which are similar to other studies^6^,^7^,^9^.

Critical incidents related to airway management have been found in 17-34% of incidents^36^,^37^ and airway management has been shown to contribute to approximately one quarter of anaesthesia related deaths^21^,^22^,^27^. In our audit respiratory causes were more frequently responsible for anaesthesia related critical incidents and mortality was mainly due to laryngospasm, hypoxia, esophageal intubation, bronchospasm and aspiration.

All anaesthesiologists aspire to an anaesthesia “system” that is completely safe. However, any system operated by human beings is subject to human failure; this is both normal and inevitable^38^. Because patterns of human error in anaesthesia as elsewhere, are identifiable predictable and repetitive, they lend themselves to classification and analysis^39^. From such analysis we gain a clearer understanding of how anaesthetists behave, which is an important step in the logical evaluation of strategies to make such failures less common.

In our audit human error has been implicated as the major cause of anaesthesia related critical incidents^3^,^4^,^15^,^32^,^35^,^40^ and mortality^8^,^33^. Lack of judgment or experience, skill and failure to check were the most frequently reported factors for human errors. Thus there are elements of human error in majority of anaesthesia related critical incidents and mortalities, although the majority of such failures were recognized and intercepted before they led to an adverse outcome. It is known that the basis for all accidents or near accidents in any situation is unsafe practice or working condition^2^.

There may have been some methodological weakness associated with our study. Firstly, under-reporting since it was based on adverse events being voluntarily reported by faculty and residents and it seems that the anaesthesiologists report major adverse events more accurately and frequently rather than minor events. Secondly critical incidents reported in this study over a one year period represent only a proportion of all mishaps that occur in association with anaesthesia resulting in a very small sample size to calculate statistical significance of risk factors.

To conclude, anaesthesia continues to be associated with mortality and morbidity despite improvements in drugs and equipments. Human error is the most important factor in the majority of these incidents. We emphasize that strategies and protocols should be developed for increasing and updating knowledge base to avoid errors of judgment. There is evidence that the use of checklists, protocols and unproved awareness of the relevance of critical incidents can improve safety^16^. Thus critical incident reporting should be introduced in all anaesthesia departments as part of quality assurance programs to ensure improved patient care.
